# Production and Characterization of a Brazil Nut Protein Isolate with Functional Properties for Food Applications

**DOI:** 10.1021/acsomega.6c04239

**Published:** 2026-09-01

**Authors:** João Lucas da Silva Rufino, Pâmela Vital da Silva de Assunção, Laura Beatriz Souza e Souza, João Pedro Cezário Brandão, Betina Raquel de Oliveira Araújo, Keyla Emanuelle Ramos de Holanda, Aníbal de Freitas Santos Junior, Edmar Vaz de Andrade, Emersom Silva Lima

**Affiliations:** † Faculdade de Ciências Farmacêuticas (FCF), Universidade Federal do Amazonas (UFAM) Av. Gen Rodrigo Otavio, n° 6200, Coroado, CEP 69077-000 Manaus, AM, Brazil; ‡ Departamento de Ciências da Vida (DCV), Universidade Estadual da Bahia (UNEB), Rua Silveira Martins, n° 2555, Cabula, CEP: 41.150-000 Salvador, BA, Brazil; § Instituto de Ciências Biológicas (ICB), Universidade Federal do Amazonas (UFAM), Av. Gen. Rodrigo Otavio, n° 6200, Coroado, CEP 69077-000 Manaus, AM, Brazil

## Abstract

Considering the nutritional properties of the Brazil nut (*Bertholletia excelsa*), a protein isolate was produced using the pH-shift precipitation technique. Analyses of the proximate composition revealed a protein concentration of 73%, 0.43% total lipids, 1.96% moisture and 6.34% total phenolic compounds. Electrophoresis revealed that this product is composed of low-molecular-weight proteins, indicating a high potential for absorption and digestion. Mineral analysis demonstrated the presence of essential minerals, especially selenium (132.88 μg/g). Amino acid analysis showed the presence of essential amino acids, particularly methionine and cysteine, which act as selenium carriers and are involved in antioxidant defense. The isolate exhibited excellent functional properties and moderate rheological properties due to the difficulty in flow caused by electrostatic forces, and the results of this study may provide support for further investigations and future applications of this product, for example in the development of nutraceuticals or pharmaceuticals based on this novel product.

## Graphical abstract

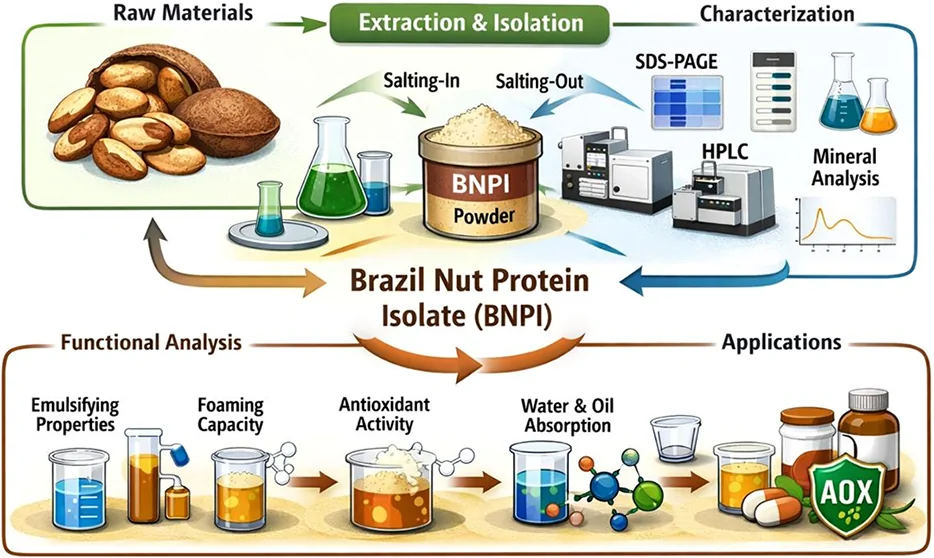

## 1 Introduction

Plant-based proteins have emerged as a promising alternative for more sustainable food production while meeting the growing global demand. They can support nutritionally adequate diets amid population growth^1^ and are particularly exemplified by textured vegetable proteins (TVP),^2^ which offer high protein content, improved nutritional quality and enhanced textural and sensory properties.

In the global search for alternative protein sources, Amazonian biodiversity offers numerous opportunities for promoting sustainable food strategies, among which the Brazil nut tree (*Bertholletia excelsa*) stands out. This species plays an important role in the regional economy and is traditionally exploited through extractive systems. Its nuts are used as a source of high–nutritional-value proteins, while the tree provides timber and fibers.^3^ Additionally, the Brazil nut tree can provide biomass for fuel production^4^ and demonstrates the ability to resprout, surviving cycles of logging and burning.^5^

Brazil nuts are rich in unsaturated fatty acids, mainly oleic and linoleic acids, as well as proteins, fibers, and minerals, with selenium being particularly noteworthy.^6^ This micronutrient contributes to the synthesis of selenoproteins, such as Se-methionine and Se-cysteine, which exhibit antioxidant activity and serve as precursors of glutathione peroxidase (GPx).^7^ Selenium has also been linked to cancer prevention through modulation of gene expression.^8^ Studies indicate that Brazil nut consumption increases plasma selenium levels, reduces oxidative stress^9,7,10,11^ and enhances GPx activity, supporting cellular antioxidant defenses^7^

Characterization analyses of Brazil nuts highlight their high nutritional quality, marked by the presence of bioactive compounds, including 24 different phenolics, a high selenium content—higher than that found in other plant-based milks—oleic and linoleic acids and several minerals.^12^ Selenium, which is also present in the protein structure of the nut, contributes to the high nutritional value of these proteins, which, once isolated, show potential for application as nutraceutical ingredients. Protein products derived from biodiversity represent promising alternatives that are capable of meeting the global demand for food in a healthy and environmentally sustainable manner, integrating technology and environmental conservation in biotechnological development.^13^

The growing demand for food and sustainability drives the development of protein isolates as viable alternatives to animal proteins, especially when derived from plants rich in essential amino acids, offering health benefits and potential for application on an industrial scale.^14^ These products have well-established uses in muscle growth promotion and recovery, in combating malnutrition, in harnessing their biological activities and in reducing environmental impact. Considering this scenario, this study aimed to characterize, in terms of its chemical, physical and physicochemical aspects, a protein isolate obtained from Brazil nuts using a protein precipitation technique based on pH differences.

## 2 Methods

### 2.1 Nut Acquisition and Processing

The nuts were obtained from an agroindustry located in the municipality of Tefé, Amazonas, Brazil. The nuts were previously refrigerated for 24 h. This step helps preserve organic compounds and facilitates removal of the kernel from the shell, allowing full utilization of the kernel without adhesion or shell residues. After this step, the kernels were dehydrated at 70 °C in a forced-air drying oven for 2 h. After cooling, the dehydrated kernels were subjected to oil extraction at room temperature using a hydraulic press. The solid residue resulting from the lipid extraction process is referred to as defatted Brazil nut cake.

### 2.2 Protein Isolate Production

The process of obtaining the protein isolate from defatted Brazil nut cake consisted of implementing and standardizing an extraction methodology focused on industrial applicability, reducing the use of chemical reagents to preserve nutraceutical quality and achieving a high protein concentration in the final product. The process is illustrated in Figure 1. Initially, the salting-in step was performed, in which the defatted cake was subjected to extraction at a ratio of 1:20 (m/v) in distilled water under agitation for 4 h at 104 °F, under alkaline conditions (adjusted to pH 9). After extraction, the system was left to stan-d for 24 h to promote phase separation. After completion of this step, the,0 precipitates with low protein content were removed, retaining only the protein-rich supernatant. Subsequently, the salting-out step was carried out, in which the pH of the solution was adjusted to 4, with the addition of NaCl at a ratio of 1 g/3 L (m/v). This change promoted the precipitation of previously soluble proteins, generating a protein-rich solid phase and a residual liquid phase with low protein content. Finally, the precipitate was dried using a spray dryer.

**Figure 1:**
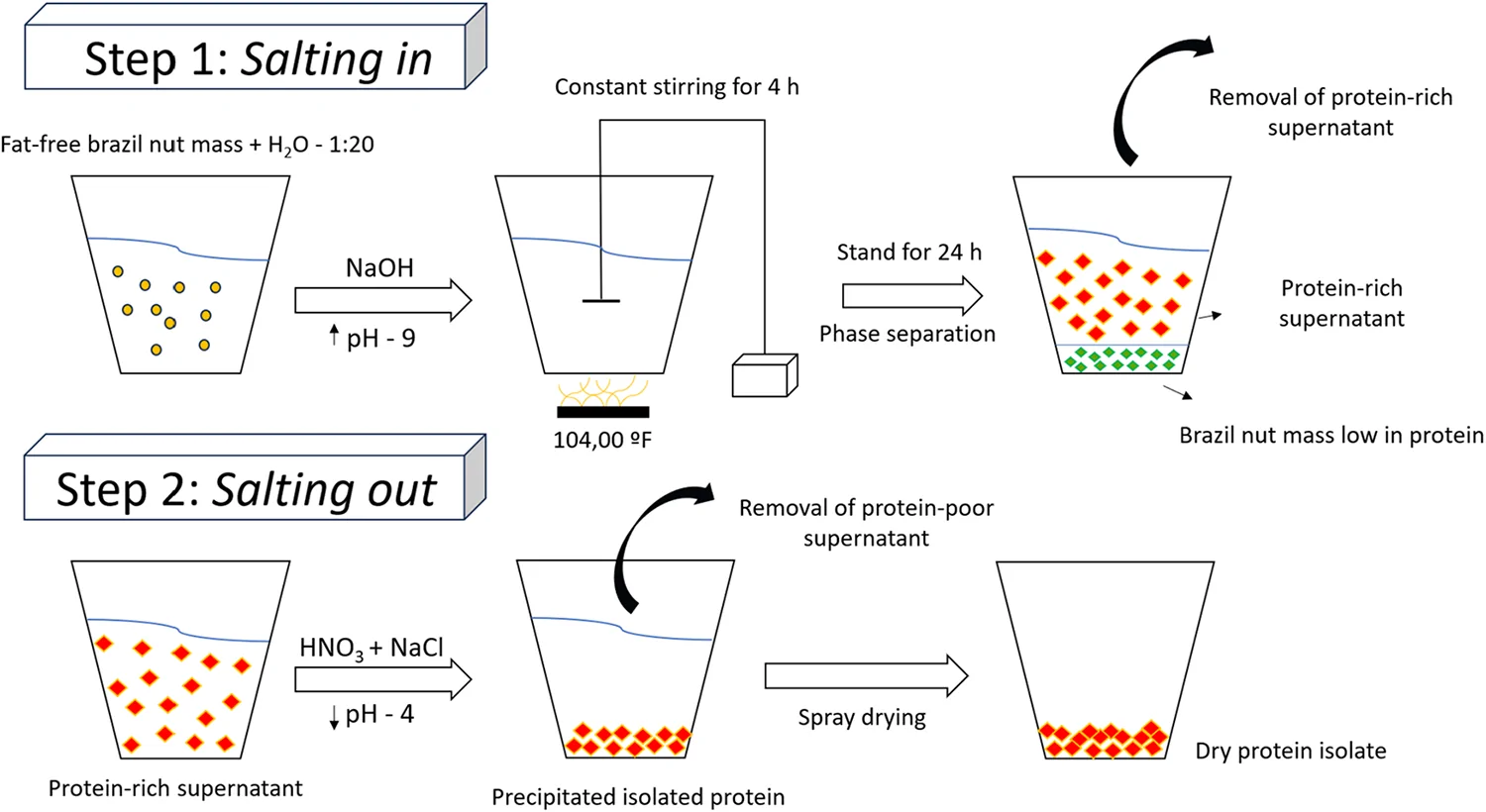
Process of extracting proteins from Brazil nuts. Protein extraction by salting: (i) Salting in, increased protein solubility at basic pH; (ii) salting out, decreased solubility at acidic pH leading to protein precipitation.

### 2.3 Proximate Composition of the Protein Isolate

Analyses of proximate composition followed protocols previously described in the Official Methods of Analysis of the AOAC (1984 and 1995) for total proteins, total lipids, moisture, carbohydrates, and ash, with adaptations.^15–17^

Protein concentration was determined using the Kjeldahl method, as described by the AOAC. Approximately 0.1 g of the sample, in triplicate, was weighed and transferred to a digestion flask, to which 1 g of catalyst (potassium sulfate + mercury oxide) and 5 mL of concentrated H_2_SO_4_ were added. Digestion was carried out under heating until complete clarification of the solution. After cooling, the digest was diluted with distilled water and treated with sodium thiosulfate solution to precipitate mercury. The solution was then alkalinized with saturated NaOH (50%) and subjected to steam-distillation, releasing ammonia (NH_4_), which was captured in a boric acid solution as an indicator. Ammonia was quantified by titration with a standard sulfuric acid solution (0.1 N). Total nitrogen content was converted to crude protein using a conversion factor of 5.46, used for Brazil nuts.

Total lipids were determined via gravimetric extraction. A 1 g aliquot of the sample was subjected to acid hydrolysis with a 4 N HCl solution, followed by extraction with hexane using a Soxhlet system. The lipid extract was dried, and its content calculated by mass difference.

Moisture content was determined via direct drying using a set of infrared scales, applying the principle of mass loss by evaporation. A 500 mg portion of the powdered sample was evenly distributed on the previously tared scales and the temperature was set to 107 °C until mass stabilization. Moisture loss was calculated as the percentage difference between the initial and final masses.

Carbohydrate content was estimated by difference. The analysis was performed by subtracting the contents of moisture, proteins, lipids, ash and dietary fiber from the total sample value. Results were expressed as a percentage relative to the mass of the original sample.

Total ash content was determined using a sample of approximately 500 mg, weighed in a porcelain crucible that had been previously calcined, cooled and tared. The material was incinerated in a muffle furnace at 600 °C for 2 h until complete elimination of organic matter. After cooling in a desiccator, the crucible was weighed and the ash content was calculated by mass difference, expressed as a percentage of the initial sample.

### 2.4 Chemical Characterization of the Protein Isolate

#### 2.4.1 Fatty Acid Determination

Oil concentration and fatty acid composition were determined using proton nuclear magnetic resonance (^1^H NMR),^18^ a method widely used for the analysis of fatty acids in edible oils. This technique is based on the fact that all fatty acid chains are esterified to a common glycerol backbone, allowing direct quantification from the ^1^H NMR spectra by comparing the integrated areas of characteristic proton signals for each fatty acid (oleic acid, ∼2.02 ppm; linoleic acid, ∼2.74 ppm; linolenic acid, ∼0.98 ppm; and saturated fatty acids, ∼0.85–0.90 ppm) with signals from the glycerol moiety.

#### 2.4.2 GC/MS Analysis

Sample preparation and analysis were carried out in two stages. The first stage consisted of an oil esterification reaction.^19^ In the second stage, the esterified oil was analyzed via gas chromatography–mass spectrometry (GC/MS) using a Shimadzu QP Plus-2010 instrument under the following conditions: an Rtx-5MS fused-silica capillary column (30 m × 0.25 mm; film thickness 0.25 μm). The injection volume (oil dissolved in dichloromethane) was 10 μL. The oven temperature program started at 100 °C (5 min) and increased at 4 °C/min to 280 °C (30 min); the injector temperature was set at 250 °C; the carrier gas was helium (linear velocity of 1.2 mL/min); injection was performed in split mode using 1.0 μL of the sample. Ionization was achieved using electron impact at 70 eV; the ion source and transfer line temperatures were 220 and 250 °C, respectively. Compound identification was based on retention times and fragmentation patterns compared with values from the NIST (National Institute of Standards and Technology) mass spectral database.

#### 2.4.3 Mineral Analysis

Samples were previously digested using a closed digestion block. Approximately 250 mg of each sample were placed directly into vessels (polytetrafluoroethylene flasks), and 7.0 mL of 65% HNO_3_ (m m^–1^) plus 1.0 mL of 30% H_2_O_2_ (m m^–1^) were added to each vessel. The heating program was carried out in four successive stages using a ramp rate starting at 75 °C (5 min), followed by 150 °C (5 min) and 200 °C (2 h), and then cooling. The digested samples were diluted with deionized water to a final volume of 45.0 mL. The resulting solutions were analyzed by inductively coupled plasma optical emission spectroscopy (ICP-OES).^20,21^ Mineral determinations were performed using a simultaneous inductively coupled plasma optical emission spectrometer (ICAP PRO XP ICP OES, Thermo Fisher Scientific, Waltham, MA), with a dual-view system, equipped with a solid-state charge injection device detector, a glass cyclonic spray chamber and a glass concentric nebulizer. Determinations were performed under the conditions recommended by the manufacturer. The spectral line for each element was selected according to criteria of absence of spectral interferences and adequate sensitivity for its determination.^22^

#### 2.4.4 Amino Acid Analysis

The amino acid profile was determined using HPLC employing a method with modifications.^23^ Samples of residues and byproducts were hydrolyzed with 6 N hydrochloric acid for 24 h. The amino acids released during this acid hydrolysis were derivatized with phenylisothiocyanate, then separated using reverse-phase HPLC and subsequently detected by UV at 254 nm.

#### 2.4.5 Total Phenolic Concentration Analysis

The technique is based on the spectrophotometric quantification of phenolic compounds using Folin–Ciocalteu reagent, a solution composed of phosphomolybdic and phosphotungstic acids, both in the +6 oxidation state. In the presence of phenolic compounds, the reagent is reduced, forming blue molybdenum and blue tungsten, with an oxidation state between 5 and 6. Absorbance was measured at 720 nm using a microplate reader (DTX 800, Beckman Coulter). The phenolic content of the samples was expressed as a percentage relative to the gallic acid standard.^24^

#### 2.4.6 SDS-PAGE Protein Electrophoresis

Initially, 1 g of Brazil nut protein isolate was solubilized in 1 mL of buffer containing 10% SDS and 100 mM Tris-HCl, pH 6.8. The sample was centrifuged at 10,000 rpm for 10 min. The pellet and lipid fraction were discarded, and the intermediate phase was used for electrophoresis analysis. Protein content was determined using the Pierce BCA Protein Assay kit (Thermo Scientific) in a microtiter plate, with 20 μL of the soluble sample. The buffer solution was used as a blank. Absorbance was measured on a microplate reader at 562 nm. The assay was performed in triplicate, and protein mass was determined from the calibration equation (*y* = 0.0652*x* + 0.172; *R* ^2^ = 0.9941). The electrophoretic profile was analyzed under denaturing and reducing conditions using 15% SDS-PAGE,^25^ with 1 μg of protein content. Electrophoresis was carried out at 200 V, 25 mA and 30 W until the migration front exited the gel (approximately 1 h). For protein band visualization, the gel was stained with Coomassie Brilliant Blue R-250 for 1 h, followed by overnight destaining in destaining solution until all excess dye was removed.

#### 2.4.7 Total Protein by Bradford

Total protein quantification was performed using the Bradford method, based on the binding of Coomassie Brilliant Blue G-250 dye to proteins, with absorbance measured at 595 nm. The standard curve was constructed using bovine serum albumin (BSA), yielding the linear equation *y =* 0.887*x +* 61.467 and a coefficient of determination (*R* ^*2*^) of 0.9952. Samples were incubated with the Bradford reagent for 5–10 min at room temperature, and protein concentration was determined by interpolation from the standard curve.

### 2.5 Physicochemical Characterization of the Protein Isolate

#### 2.5.1 Functional Properties of the Protein Isolate

The analysis of the functional properties of the protein isolate was adapted^26^ for each of the properties described below:

(a) Water and oil absorption capacity: To determine water and oil absorption capacity, 1 g of the sample was weighed and dispersed in distilled water and vegetable oil at a ratio of 1:10 (m/v). After mixing and centrifugation, the tube containing the sample was weighed, and the difference in weight between the dry sample and the sample with water or oil was calculated.
(b) Foam formation and stability: Foam-forming capacity and stability were determined using aliquots of protein isolate at a concentration of 2% (m/v), dispersed in 25 mL of distilled water. The pH was adjusted to 1.0, 3.0, 5.0, 7.0 and 9.0, and the samples were then homogenized at 12,000 rpm for 1 min. The volume of the foam formed at the end of 1 min of homogenization represented the foam-forming capacity. Foam stability was evaluated by measuring the decrease in volume relative to the initial volume at time intervals of 5, 10, 30, 60, and 120 min.
(c) Emulsifying capacity and stability: Emulsifying capacity and stability were determined by analyzing a sample suspension (sample of 50 mg dispersed in 5 mL of distilled water) homogenized with vegetable oil (5 mL) to form emulsions. Prior to centrifugation (3,200 rpm for 5 min), the emulsion samples were heated at 80 °C for 30 min to assess emulsion stability. The samples were adjusted to pH 1.0, 3.0, 5.0, 7.0, and 9.0 to evaluate their behavior under different pH conditions.

#### 2.5.2 Water Activity (aW)

This assay was performed using a water activity analyzer with an adjustable laser diode (AquaLab 4TE) according to the manufacturer’s instructions. Three grams of each sample were analyzed, with the method based on the chilled-mirror dew point technique. Water activity was measured directly based on the equilibrium between the relative humidity of the air and the vapor pressure of the sample. Results were expressed as water activity values.

### 2.6 Physical Characterization of the Protein Isolate

#### 2.6.1 Thermogravimetric Analysis (TG/DTG and TG/DTA)

The thermal properties of the protein isolate were analyzed using TG/DTG (thermogravimetry – derivative thermogravimetry) and TG/DTA (thermogravimetry – differential thermal analysis).^27^

The thermogravimetric analysis aimed to characterize the thermal profile and mass changes, such as gains or losses, of the DCHA. The procedure was carried out on a DTG-60 thermobalance (Shimadzu, Japan), using a sample of approximately 10 mg, heated from 25 °C to 1,000 °C, at a heating ramp rate of 10 °C min^–1^ under a nitrogen atmosphere (99.98%, White Martins, Brazil), with a flow rate of 50 mL.min^–1^.

#### 2.6.2 Rheological Properties of the Powder: Repose Angle, Bulk and Tapped Density, Powder Particle Size, and Flow Rate

The rheological properties of the protein isolate were analyzed, for characterization of its morphology. To determine the repose angle, the fixed-funnel technique was used, in which 50 g of the protein isolate was poured through a funnel positioned at a constant height over a flat surface. After stabilization of the resulting cone, its height and diameter were measured, and the result was expressed in degrees (°) as an indicator of flowability.^28^

Bulk density was determined by placing a 50 g sample into a 100 mL graduated cylinder without compaction. The occupied volume was recorded, and bulk density was calculated. For tapped density, the same cylinder was subjected to 500 mechanical taps. After volume stabilization, the tapped density was calculated. Values were expressed in g/mL.

Particle size analysis was performed by mechanical sieving, using a set of standardized sieves with mesh sizes ranging from 2,000 to 125 μm. A sample of approximately 50 g was placed on top of the sieve stack and subjected to agitation for 10 min. After the analysis period, the material retained on each sieve was weighed, and the results were expressed as a percentage of the sample’s mass, allowing for particle classification and distribution (Figure 2).

**Figure 2:**
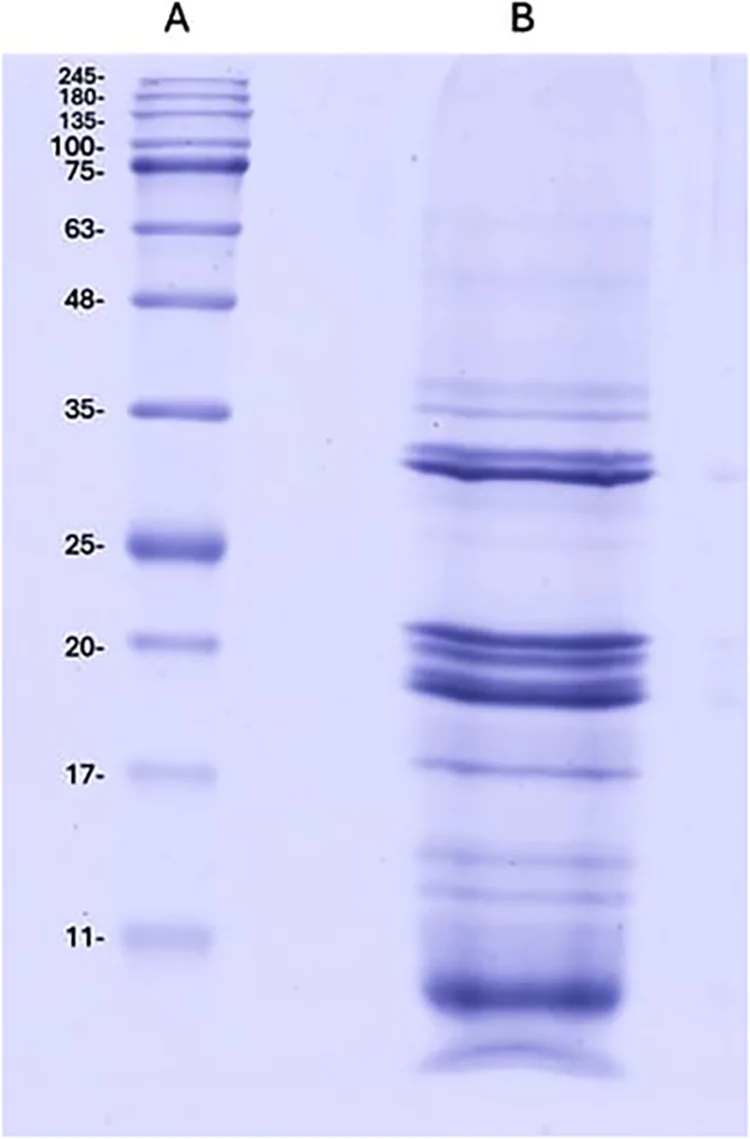
SDS-PAGE of the protein isolate. A = BlueEye Prestained Protein Marker; B = Protein isolate.

#### 2.6.3 Scanning Electron Microscopy (SEM)

The samples were mounted on metal stubs using conductive carbon tapes and then coated with gold (approximately 15 nm thickness) using a smart coater (JEOL, DII-29010SCTR). Subsequently, analyses were performed on a scanning electron microscope (JEOL, JSM-IT500H) operating in secondary electron mode.^29^

## 3 Results

### 3.1 Protein Isolate Extraction

Table 1 shows the parameters analyzed during the standardization of the protein extraction process. The results are presented in two phases: salting in and salting out. In the first phase, it was observed that pH 9 extracted the most protein from the analyzed water; then, at pH 9, an extraction time of 4 h showed the best cost-benefit ratio; finally, at pH 9 and with a 4 h extraction, an average temperature of 104 °F proved to be the most efficient for protein extraction. In the second phase, pH 4 was able to precipitate the highest amount of protein in the analyzed supernatant.

**Table 1:** Variables Analyzed During the Standardization Stage of Obtaining the Protein Isolate, Both in Salting In and Salting Out, of Brazil Nuts^a^

salting in						salting out	
pH	concentration mg/mL	pH 9/time h	concentration mg/mL	pH 9/4 h/T° °F	concentration mg/mL	pH	concentration mg/mL
8	1.413 ± 0.12	1	1.270 ± 012	77	1.151 ± 0.12	5	0.179 ± 0.08
**9**	**1.475 ± 0.08**	2	1.445 ± 0.08	**104**	**1.191 ± 0.09**	**4**	**0.056 ± 0.09**
10	1.419 ± 0.09	3	1.412 ± 0.07	158	1.067 ± 0.07	3	0.422 ± 0.09
11	1.364 ± 0.11	**4**	**1.660 ± 0.12**			2	0.668 ± 0.12
12	1.369 ± 0.12	5	1.676 ± 0.10			1	0.640 ± 0.11
		6	1.670 ± 0.11				

a Concentration values calculated using the Bradford method with *R* ^2^= 0,9952.

### 3.2 Proximate Composition of the Protein Isolate

The result for total phenolic compound analysis was 6.32% ± 0.005, while the proximate composition of the Brazil nut protein isolate is presented in Table 2 below.

**Table 2:** Centesimal Composition of the Protein Isolate^a^

centesimal composition	protein isolate (%)
total proteins	73.0 ± 2.00
total lipids	0.43 ± 0.25
moisture	1.96 ± 0.16
carbohydrates	20.23 ± 0.20
ashes	6.34 ± 0.06

a The result are expressed as a percentage.

### 3.3 Chemical Characterization of the Protein Isolate

#### 3.3.1 Characterization of the Oil Extracted from the Protein Isolate

The results of the lipid composition analysis of the protein isolate are presented in Table 3. The percentages refer to the total of 0.43% total lipids present in the sample after the drying process.

**Table 3:** Lipidic Characterization

lipid composition	protein isolate (%)
linoleic acid	29.51 ± 0.5
oleic acid	43.29 ± 0.5
saturated fat	26.91 ± 0.5

#### 3.3.2 Mineral and Amino Acid Analysis

The mean values for mineral and amino acid content are presented in Table 4 below.

**Table 4:** Mineral Analysis and Amino Acid Composition

minerals	concentration μg/g	amino acid	concentrationmg/kg
**Cr**	0.50 ± 0.02	**TRIP**	1.90 ± 0.01
**Cd**	0.03 ± 0.02	**ASP**	12.8 ± 0.01
**Cu**	81.3 ± 0.02	**SER**	8.40 ± 0.01
**Pb**	0.26 ± 0.02	**GLU**	35.4 ± 0.04
**Ni**	6.50 ± 0.02	**GLY**	8.00 ± 0.01
**Mn**	5.00 ± 0.02	**HIS**	3.80 ± 0.01
**Fe**	38.8 ± 0.02	**ARG**	28.4 ± 0.20
**Co**	2.80 ± 0.02	**THR**	3.90 ± 0.04
**Zn**	9.40 ± 0.02	**ALA**	5.40 ± 0.01
**Ag**	0.03 ± 0.02	**PRO**	8.40 ± 0.01
**Sb**	0.02 ± 0.02	**TYR**	6.30 ± 0.00
**Al**	39.2 ± 0.02	**VAL**	7.60 ± 0.01
**Ca**	472.7 **±** 0.02	**LYS**	4.50 ± 0.01
**Ba**	313.2 ± 0.02	**ILE**	5.30 ± 0.05
**K**	5,884.9 ± 0.02	**LEU**	13.0 ± 0.01
**Na**	3,536.3 ± 0.02	**PHE**	8.10 ± 0.01
**Mg**	1,375.6 ± 0.02	**CIS**	5.20 ± 0.00
**Se**	132.88 ± 0.02	**MET**	21.9 ± 0.01

#### 3.3.3 SDS-PAGE Electrophoresis of the Protein Isolate

The polyacrylamide gel image from the electrophoretic run is shown below, revealing a higher concentration of low-molecular-weight proteins.

### 3.4 Physicochemical Characterization of the Protein Isolate

#### 3.4.1 Functional Properties

Table 5 and Figure 3 present the data from the physicochemical analyses of the protein isolate.

**Figure 3:**
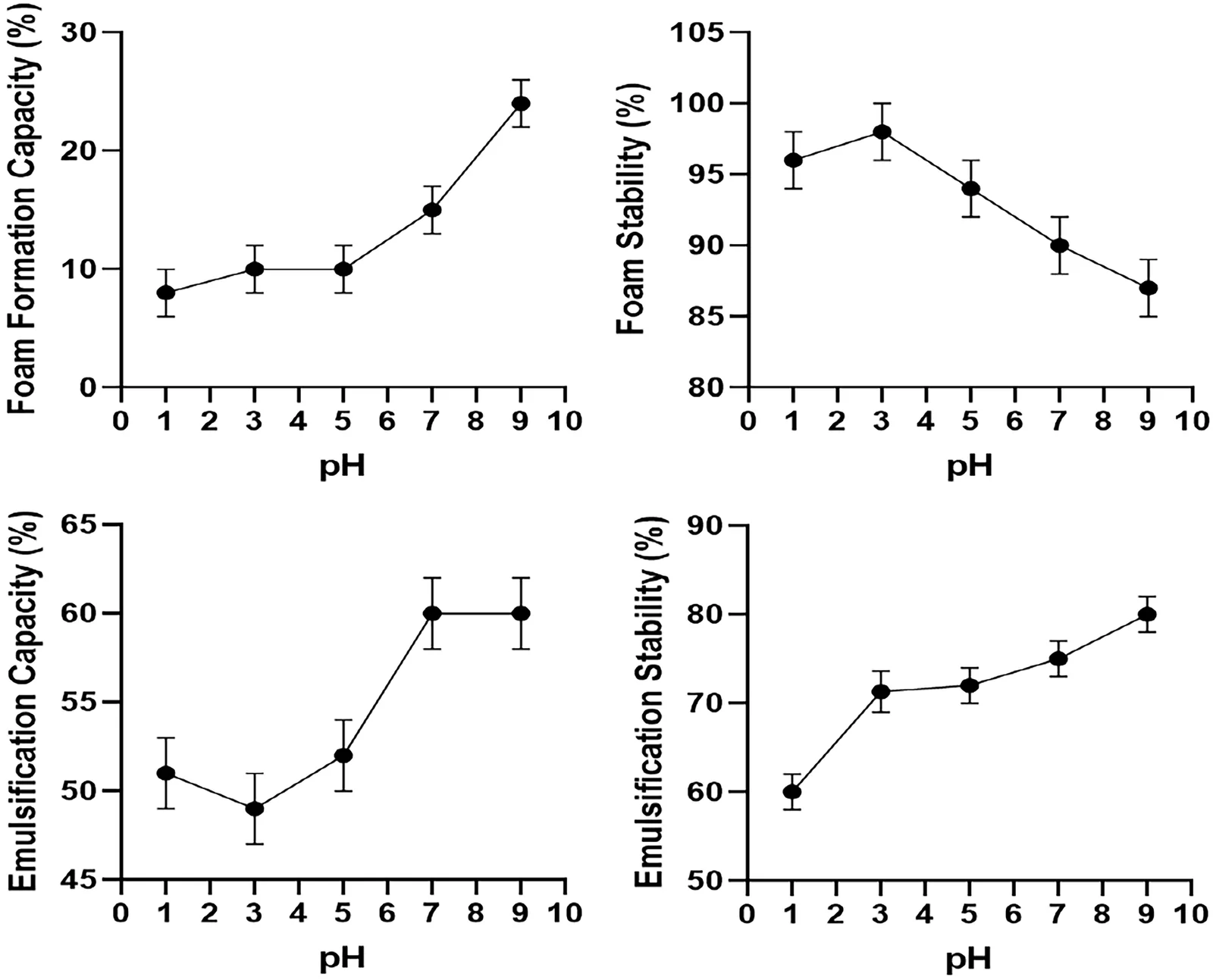
Emulsification and foam formation capacity and stability at different pHs.

**Table 5:** Functional Properties

functional properties	protein isolate (%)
water absorption capacity	65 ± 2
oil absorption capacity	145 ± 2
AW	0.24 ± 0.01

### 3.5 Physical Characterization of the Protein Isolate

#### 3.5.1 Thermogravimetric Analysis TG/DTG and TG/DTA

The results of the TG/DTG and TG/DTA analyses are presented in Figure 4 below.

**Figure 4:**
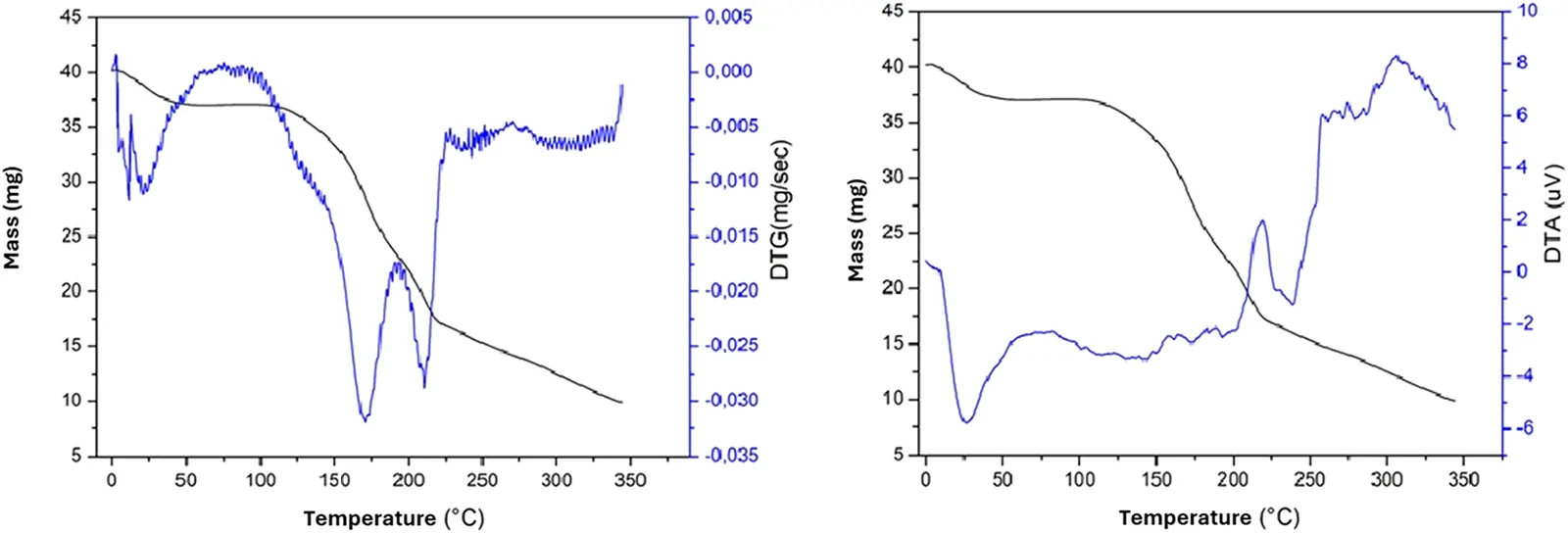
TG/DTG and TG/DTA graph of the Brazil nut protein extract

#### 3.5.2 Analysis of the Rheological Properties

The rheological characteristics of the powder obtained after spray drying showed a repose angle of 24° ± 1, a bulk density of 0.294 ± 0.01 g/mL, a tapped density of 0.526 ± 0.01 g/mL, in a measured flow rate.

#### 3.5.3 Scanning Electron Microscopy (SEM)

The results of the scanning electron microscopy are presented in Figure 5. It can be observed that the particles exhibit a size distribution that is not uniform.

**Figure 5:**
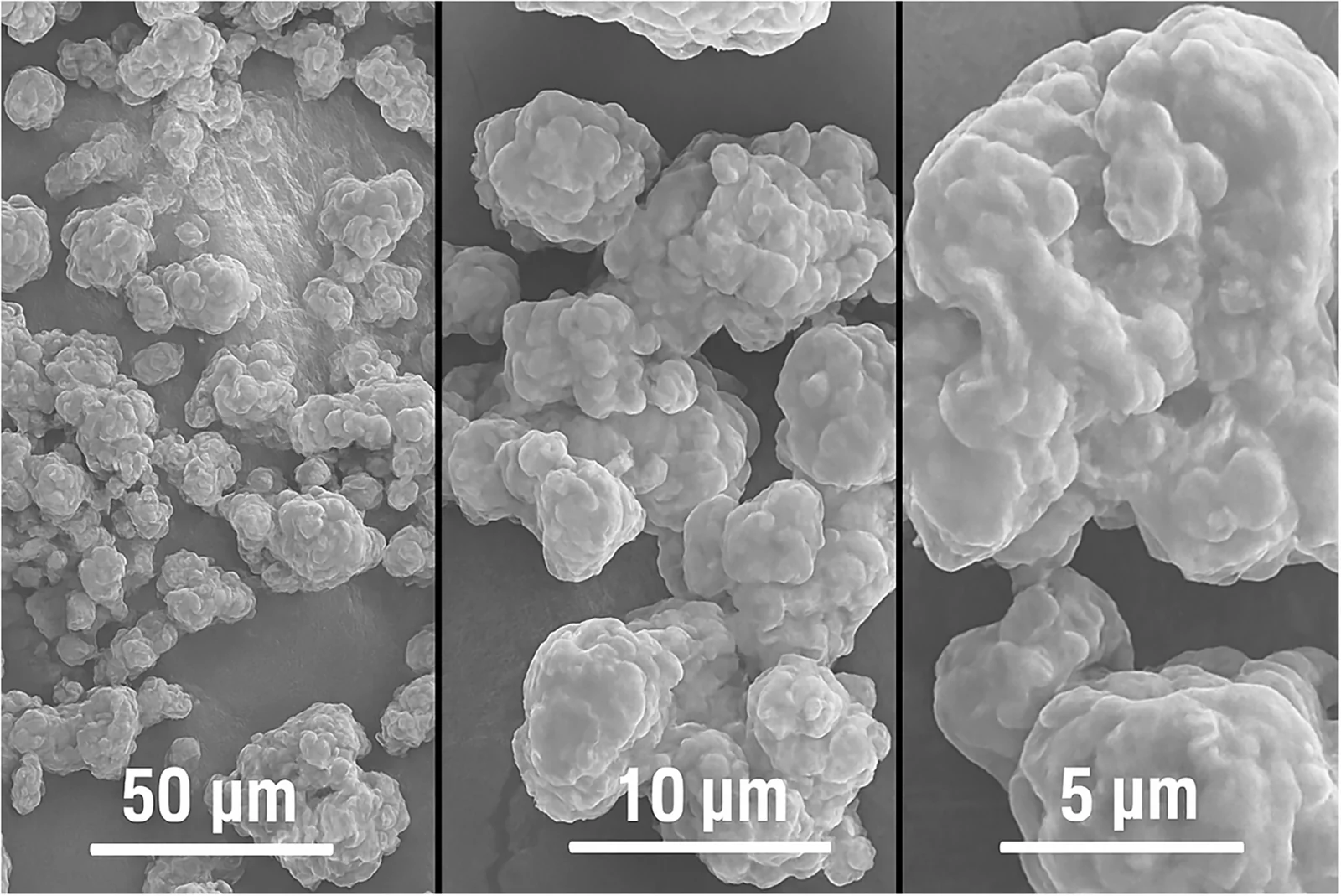
Scanning electron microscopy of Brazil nut protein isolate. Irregular structures with significant size variability are observed at different magnifications. From left to right, the images progress from a broader field at 50 μm to increasingly detailed views at 10 and 5 μm, highlighting the morphological complexity of the sample.

## 4 Discussion

Using defatted Brazil nut cake at a 1:20 nut/distilled water ratio, with pH adjustment (NaOH) to basic levels under agitation, the proteins in the cake begin to migrate into the water. This process is known as salting-in, in which ions resulting from NaOH dissociation enhance the solubilization of protein macromolecules.^30^ This protein-rich supernatant is subjected to pH adjustment (HNO_3_) to acidic levels, with the addition of salt (NaCl), to initiate protein precipitation. This process is known as salting-out, in which ions resulting from the dissociation of HNO_3_ and NaCl reduce the solubilization capacity of protein macromolecules.^30^

Nitric acid was selected because it provides efficient and reproducible pH adjustment during the precipitation stage. In this process, the acid acts only as a processing aid and not as a functional ingredient of the final isolate. Nevertheless, food-grade organic acids, such as citric acid, could be evaluated in future studies focused on clean-label applications and industrial scale-up.

Guided by this principle, variables during the extraction process were adjusted to increase protein extraction efficiency. At basic pH levels, five different pH ranges from 8 to 12 were tested, and it was observed that pH 9 yielded the highest protein extraction, with the highest values shown in Table 1. Using pH 9, six different extraction times ranging from 1 to 6 h were tested, and it was observed that 4 h of extraction provided the best cost-benefit ratio based on the protein concentration in the analyzed supernatant.

Carrying out the extraction at pH 9 for 4 h, the influence of temperature during the process was tested at three levels—77 °F, 104 °F, and 158 °F—and it was observed that 104 °F resulted in the highest protein concentration in the supernatant. After completing the salting-in step, the process proceeded to salting-out, where the influence of acidic pH was evaluated across different ranges, from 5 to 1, together with NaCl at a ratio of 1 g/L of supernatant.

At pH 4, the best precipitation results were observed, yielding a lower protein concentration in the supernatant. The precipitated material was then subjected to spray drying. The spray-drying process produces more uniform protein particles, making it a highly efficient method for preserving organic compounds.^31^ The material obtained after extraction was designated as the “protein isolate.” With the protein isolate in hand, the characterization steps of this product were then carried out.

For the proximate composition, determination of protein concentration was performed first. The average protein concentration of 73% indicates a high protein purity in the final isolate. This value represents the proportion of protein in the dried material, which was achieved without the use of chemical reagents—only for pH control—making it suitable for human consumption and use in food formulations. The Brazil nut is a source rich of protein, especially in the form of defatted Brazil nut cake.^6^

Brazil nut proteins are formed under unique conditions in an extremely lipophilic environment, as the nut is a lipid-rich product. Even after extraction under hydrophilic conditions, an average of 0.43% total lipids was still obtained. Due to the functional properties of proteins, intermolecular interactions may occur, such as water- and oil-holding capacity,^32^ resulting in the coextraction of a lipid fraction even under hydrophilic extraction conditions.

The drying process directly influences the final moisture content of the product. This factor is important for the microbiological and biochemical stability of the food. High water content and its associated nutritional values can promote microbial contamination and also lead to deterioration through hydrolysis reactions, as well as changes in texture, appearance and color. Since food safety is one of the primary concerns in industrial food development due to the risk of foodborne illnesses,^33^ the moisture content of 1.96% is considered satisfactory to help prevent such occurrences.

The presence of carbohydrates at a concentration of 20% gives the Brazil nut protein isolate a more functional, balanced and appealing profile for consumption. There is a potential for rapid energy supply to the body, especially in high-protein diets, which may help prevent muscle fatigue, while also improving texture, solubility and flavor. Additionally, many carbohydrates can be associated with dietary fibers.^34,35^

The determination of total ash in protein products, such as protein isolates, is a fundamental parameter for mineral characterization of the product. Considering the mineral analysis data, which show that this isolate contains all essential minerals for human health, a concentration of 6.34% indicates a strong mineral content, even compared to other products. These elements are essential for physiological functions such as enzyme regulation, maintenance of bone integrity, oxygen transport, antioxidant activity and electrolyte balance. This quantification confirms the nutritional quality of the protein isolate, clearly highlighting its potential for use in functional food formulations, nutritional supplements and therapeutic applications.

Although the determined value for total phenolic compounds is low, averaging 6.32%, the nature of the product may justify this percentage. Phenolic compounds are widely distributed in plant species, such as in *Bertholletia excelsa*, however, their concentrations are generally lower in protein-rich fractions or protein isolates than in whole plant tissues or other nonprotein fractions.^36^ Even at low concentrations, the presence of phenolics suggests an antioxidant nature of the product.

The average total lipid content was 0.43% Although present in low amounts, its composition is of particular interest. With an average linoleic acid content of 29.51% and oleic acid at 43.26%, a total of 72.77% of compounds capable of reducing serum levels of inflammatory markers,^37^ as well as contributing to antioxidant defense and prevention of tissue damage,^38^ is observed. The intrinsic lipid composition of the protein isolate is nutritious, significant, and contributes to an overall enhancement of the product’s nutritional quality.

Many minerals act as enzyme cofactors, which support the performance of biochemical reactions. For example, selenium (Se) is present as a selenocysteine residue in glutathione peroxidase, replacing sulfur and forming a selenoprotein,^39^ which serves as a basis for the formation of antioxidant compounds, potentially contributing to the intracellular antioxidant defense system. Among various nuts, the Brazil nut has been highlighted as one of the richest sources of bioavailable selenium.^40^

The selenium content observed in the protein isolate is notably high when compared to the recommended daily intake for adults (55 μg/day). This indicates that relatively small amounts of the isolate could provide a substantial contribution to daily selenium requirements. However, it is important to consider that selenium availability is influenced by digestion and absorption processes. Therefore, despite the elevated concentration, the effective nutritional contribution of selenium from this isolate remains dependent on its bioaccessibility and the physiological conditions of the gastrointestinal tract, which may limit its actual uptake.^41^

The presence of calcium (Ca), iron (Fe), magnesium (Mg), manganese (Mn) and zinc (Zn)—metals considered essential for the human body^42^ confers a high nutritional quality to the protein isolate due to their amounts per gram, as shown in Table 4. It is well-known that calcium is involved in the formation and maintenance of bones and teeth, iron plays a role in hemoglobin formation and oxygen transport, magnesium is associated with muscle contractions and regulation of heart rhythm, manganese is related to lipid, carbohydrate and protein metabolism, and zinc is important for the immune system. Low intake of these minerals can lead to serious health effects, such as hyponatremia, diarrhea, adrenal gland disorders and potential impairment of the physiological functions of each mineral in the body.

Although antinutritional factors were not quantified in the present study, the aqueous extraction, phase separation and protein precipitation steps may contribute to the partial removal of water-soluble nonprotein constituents. Nevertheless, further studies are required to determine the phytic acid content of the isolate and its potential impact on mineral bioavailability.

Initially, the protein isolate can readily serve as a mineral source for replenishment in the body, as it contains high levels of essential minerals, contributing to the homeostasis of various biochemical processes.

Amino acid analysis is important for evaluating the structural composition of these proteins, as amino acids are involved in the synthesis of proteins that make up muscles, tendons, cartilage and other tissues, as well as participating in the regulation of cellular transport mechanisms.^43^ The method used assessed the presence of 18 amino acids, as shown in Table 4.

The protein isolate contains all the amino acids that are considered essential for the diet. The highest concentrations were found for arginine, methionine and glutamine, amino acids involved in wound healing, immune system support, muscle mass gain and the cellular antioxidant defense system.^44^ When compared to other protein isolates obtained from the same species, the levels of glutamine and arginine are notably higher.^45^

Glutamine and arginine, individually, have significant capacities to protect the intestinal mucosa against adverse effects of chemotherapy and radiotherapy, as they help prevent methotrexate-induced disruption of the intestinal barrier by regulating claudin-1, likely through ERK and NF-κB pathways.^46^ It is therefore suggested that incorporating this protein isolate, with its high content of these amino acids, into the diet may help improve clinical outcomes by mitigating the adverse effects of such treatments.

Furthermore, with cysteine and methionine combined with the high selenium levels in the protein isolate, it can be inferred that amino acids capable of supporting the synthesis of proteins and liver detoxifying compounds are present, as well as selenated forms that can deliver this mineral to the body’s antioxidant pathways, resulting in protection against oxidative stress, maintenance of homeostasis and neutralization of oxidative damage to cell membranes.^47^

The procedure for obtaining the protein isolate reveals further peculiarities in electrophoresis. As shown in Figure 2, the predominant proteins in this product range from <11 kDa to 35 kDa.^48^ In the analysis of isolated Brazil nut proteins by another method, the presence of proteins up to 75 kDa was reported,^12^ contrasting with the data from this study, which also showed a major composition of albumins and globulins. Thus, it can be observed that our method prioritizes the extraction of proteins that provide low-molecular-weight subunits.

With proteins ranging in molecular weight from 0–35 kDa, it is suggested that this protein isolate is extremely rich in low-molecular-weight proteins, which have become highly relevant in recent years due to their ease of absorption^47^ and favorable kinetics related to digestive processes. These results therefore indicate a high likelihood of the presence of albumins, particularly 2S albumin in the 10 kDa range, and globulins concentrated around 20 kDa,^12,49^ as well as several peptides below 10 kDa.

Albumins have excellent biocompatibility, the ability to cross biological barriers (rapid absorption), enzymatic activity, a stable half-life, and also serve as antioxidants.^50^ Globulins function in substance transport, antibody formation, enzymatic activity, involvement in the blood coagulation cascade, energy provision, and exhibit notable functional properties by improving texture and stabilizing dispersions.^51^

Peptides are short chains of amino acids that serve as the building blocks of proteins, although many also occur as independent bioactive molecules. They are formed through peptide bonds linking two or more amino acids and may exhibit metal-binding or chelating properties. As next-generation therapeutics, peptides occupy an intermediate position between small molecules and macromolecules, yet often surpass both in biochemical and therapeutic potential by acting as signaling agents in biological processes and as components of cellular defense mechanisms.^52^ Thus, the presence of proteins and peptides with enhanced absorption capabilities and potential biological activities, along with peptides involved in mineral transport within the body, increases the nutraceutical quality of the Brazil nut protein isolate.

The presence of low-molecular-weight proteins in plant-derived protein isolates may raise important considerations regarding allergenic potential. These proteins, often characterized by high solubility and structural stability, can include seed storage proteins and enzymatic fractions that are resistant to thermal processing and proteolytic digestion. Such properties may favor their persistence during gastrointestinal digestion, increasing the likelihood of interaction with the immune system and, consequently, allergenic responses in susceptible individuals. Although the primary protein fractions in this study were not specifically identified as known allergens, the potential presence of low-molecular-weight proteins suggests the need for further characterization, particularly in the context of food and nutraceutical applications, where allergenicity remains a critical safety parameter.^53^

Interest in plant proteins as an alternative to animal proteins has grown rapidly due to their lower cost, health benefits and superior functional properties. The functional properties of proteins are essential for their applications in foods. They act as gelling agents, thickeners, foaming agents and emulsion stabilizers, providing not only nutritional value but also sensory attributes such as color, flavor and texture.^54^

Functional properties determine the applicability of any protein within the food sector. For example, the oil and water retention capacities of proteins are essential in the development of meat and bakery products, while their emulsifying properties are fundamental in the production of ground meat, nondairy cakes and desserts.^55^

According to the data in Table 5 and Figure 3, the lowest values were observed for foam-forming capacity, ranging from 8–24%, yet it showed a high ability to maintain foams, at 87–96%, with stability decreasing as pH increased. These results suggest that food formulations containing this protein isolate could develop denser textures. In contrast, the observed emulsifying capacity of 49–60% and emulsion-stabilizing capacity of 60–80%, which vary with increasing pH, indicate strong applicability in the formulation of textured food products across a wide pH range, since coagulation and phase separation events tend to be mitigated as emulsion stability increases.

A water absorption capacity of 65% and an oil absorption capacity of 145% indicate properties that are essential for incorporation into meat products, as many of these foods consist of both aqueous and oily matrices arranged in an integrated manner. These results also suggest a possible explanation for the coextraction of a lipid fraction (7.03%) together with the proteins during the aqueous extraction process.

To analyze the thermal behavior of the protein isolate, thermogravimetric analysis and its derivative were performed. The isolated protein is heated in a controlled atmosphere, allowing monitoring of mass loss as a function of temperature. The DTA curve (Figure 4) provides information on thermal stability and physical or chemical transformations that occur during heating, while the DTG curve (Figure 4) highlights thermal inflection points corresponding to the maximum rates of mass loss.

The results of the TG/DTG curves can be observed in Figure 4. Based on the obtained derivatives, four stages of decomposition of the Brazil nut protein extract can be inferred. The first stage occurs at temperatures below 100 °C, with a mass loss of analytical concentration (Ca) of 6.25%, corresponding to the evaporation of volatile substances and physically adsorbed water^56^ on the surface of the protein extract. This loss is accompanied by an endothermic event, as can be observed in the TG/DTA results (Figure 4).

The second degradation event of the sample was observed before reaching 150 °C. Based on the derivative and the energy involved in this event, a mass loss of Ca of 20% was recorded, accompanied by an exothermic event, which may be associated with the decomposition of sugars present in the studied matrix.^57,58^ The third mass loss was observed at approximately 200 °C, Ca of 41.6%, accompanied by an exothermic event. This event is more likely associated with the thermal degradation of the protein backbone and complex protein–carbohydrate interactions present in the sample.^57^ Finally, the fourth mass loss occurred from 225 °C, Ca of 42.8%, with an exothermic event associated with the last stage of the analysis. This degradation can also be attributed to advanced thermal breakdown of protein structures and associated macromolecular interactions within the matrix.

Analyzing the rheological properties of the powders is important for pharmaceutical design, as it helps ensure processing efficiency and the quality of the industrial products. The spray-drying method improves drying efficiency and reduces energy costs during production,^59^ while also ensuring particle uniformity. Therefore, it is closely related to the potential processing qualities for industrial applications.

After the procedures to assess the particle size of the protein isolate powder, it was observed that it is a fine powder. Although it has this characteristic, it does not flow freely. Proteins are held together by electrostatic forces, which promote particle aggregation, creating the need for agents that can improve flow, prevent clumping and increase stability and versatility in other pharmaceutical applications. For example, colloidal silica is a glidant capable of adhering to the particle surface, disrupting the action of these electrostatic forces between particles.^60^

Apparent bulk density, which accounts for both the powder mass and its porosity, is used to determine the appropriate capsule size for a potential formulation, as well as the need for additional diluent agents.^61,62^ Tapped density, on the other hand, reflects a powder’s ability to be compacted, as materials with good compressibility are generally easier to process industrially and also influence disintegration and hardness.^63^ The observed results indicate good compressibility and adequate porosity for the incorporation of pharmaceutical excipients into the formulation.

Nutraceutical characterization analyses are important for providing data that serve as a basis for incorporating the substance into the food industry items. Plant proteins represent a new perspective in the food sector.

When comparing the data from this study with an evaluation of a hazelnut protein isolate regarding its technological and physicochemical properties, the results reinforce the nutritional quality of hazelnut protein and its behavior across different pH ranges.^64^ Hazelnut protein exhibits better foam-forming behavior at very acidic pH levels. This indicates its ability to structure and maintain texture in highly acidic foods; however, the ability to maintain a pleasant texture decreases as pH gradually increases, potentially resulting in a denser and heavier texture.

With an emulsification capacity slightly above 50%, even small temperature changes can result in phase separation and coagulation. The average hazelnut protein content ranged from 81–86%, although the remaining composition was not reported. Although the data for this hazelnut protein isolate are similar to those obtained for the Brazil nut isolate, the differences highlight a broader range of applications for Brazil nut protein as well as a more pronounced nutritional quality. Even with a slightly lower average protein concentration of 73%, the remaining 27% of the product has antioxidant and anti-inflammatory properties.^65^

In a cashew nut protein isolate, functional and technological properties were evaluated, providing data on the technological application potential of this isolate. The extraction method used methanol as the solvent; however, the drying method was not reported. In this context, details regarding particle structure remain unavailable for interpretation. The values found for the set of functional properties of cashew nut protein were low, and did not reach at least 30% for emulsification capacity, for example, which indicates a somewhat more limited product for application in food formulations compared to our Brazil nut protein isolate.^66^

Although the oil absorption capacity is high, the cashew nut protein isolate apparently cannot produce emulsifying systems and soft, light textures without the aid of additional excipients. The extraction and drying method of this protein isolate may also have influenced the retention of its functional properties, since chemical reagents were used, unlike the method for obtaining the Brazil nut protein isolate, where only subtle pH adjustments are made in an aqueous extraction system.

When compared with commonly used commercial protein ingredients such as soy and pea protein isolates, the Brazil nut protein isolate presents a competitive functional profile, particularly regarding oil absorption and emulsion stability. While soy protein generally exhibits superior emulsifying performance due to its higher purity and extensive industrial optimization, the Brazil nut isolate offers the additional advantage of naturally occurring selenium and sulfur-containing amino acids, which may contribute to its nutritional differentiation in the plant protein market.^67^

The heterogeneous morphology observed in the micrograph of the Brazil nut protein isolate, with particles of irregular sizes and contours, is consistent with protein systems in which conditions favor electrostatic interactions with residual charges that alter the ζ-potential, thereby changing the balance between electrostatic repulsion and attractive forces. The system approaches the isoelectric point, favoring particle aggregation, which is a direct consequence of this electrostatic regime in the protein system. This governs the aggregation kinetics and particle architecture, resulting in variability in size and shape.^68,69^

The present study provides a comprehensive compositional characterization of a protein isolate obtained from Brazil nuts using a protein precipitation method based on pH differences. In addition to the pronounced nutritional quality of the isolate, it was identified that the product can be incorporated into a wide range of foods, across pH levels from acidic to mildly basic, as well as pharmaceutical formulations, due to its excellent density characteristics. The addition of glidants may be required to improve flow during industrial processing, and the isolate demonstrates resistance to temperature increases during processing. The method used to obtain the protein isolate can influence the retention of the product’s nutritional properties, distinguishing it in terms of quality compared to isolates from other nuts. Thus, the results of this study can provide a basis for further research and potential applications of this nutraceutical.

## Supplementary Material



## Notes

1H NMR spectrum of oil extracted from Brazil nut protein isolate; and GC/MS chromatogram and compound identification of oil extracted from Brazil nut protein isolate (PDF)

## References

[1] Garza-Caballero S., Heredia-Olea E., Tejada-Ortigoza V. (2025). Development and characterization
of texturized vegetable proteins with faba bean, sesame, and amaranth
for protein quality improvement. Food Chem..

[2] Auppasuk P., Chaisuwan B., Ploypetchara T. (2025). Influence of elevated
protein content in textured vegetable protein on the textural characteristics
and sensory attributes of plant-based meat alternatives. Appl. Food Res..

[3] Salomão R. P., Goeldi M. P. E. (2014). A castanheira:
história natural e importância
socioeconômica. Cienc. Nat. Belém..

[4] Colpani D., Santos V. O., Lima V. M. R. (2023). Improving biomass fuel
obtained from Brazil nut residues via torrefaction: A case of kinetic
and thermodynamic study. J. Anal. Appl. Pyrol..

[5] Paiva P. M., Guedes M. C., Funi C. (2011). Brazil nut
conservation through shifting
cultivation. Forest Ecol. Manage..

[6] Santos O. V., Correa N. C. F., Carvalho Jr R. N. (2013). Comparative parameters
of the nutritional contribution and functional claims of Brazil nut
kernels, oil and defatted cake. Food Res. Int..

[7] Li, Y. ; Clark, C. ; Abdulazeeme, H. M. The Effect of Brazil Nuts on Selenium Levels, Glutathione Peroxidase, and Thyroid Hormones. In A Systematic Review and Meta-Analysis of Randomized Controlled Trials; Journal of King Saud University. Science Direct, 2020; pp 1845–1852.

[8] Othman F. B., Mohamed H. J. B. J., Sirajudeen K. N. S. (2017). The influence of selenium
status on body composition, oxidative DNA damage and total antioxidant
capacity in newly diagnosed type 2 diabetes mellitus: A case-control
study. J. Trace Elem. Med. Biol..

[9] Rae, C. D. ; Williams, S. R. Glutathione in the human brain: Review of its roles and measurement by magnetic resonance spectroscopy. In Anal. Biochem.; Elsevier, 2017; pp 127–143. 10.1016/j.ab.2016.12.022 28034792

[10] LI J., Zhang W., Zhou P. (2022). Selenium deficiency
induced apoptosis via mitochondrial pathway caused by Oxidative Stress
in porcine gastric tissues. Res. Veterinary
Sci..

[11] HIRSCH, S. Selenium Deficiency is Associated with Polyneuropathy in Primary Sjögren’s Syndrome; Clinical Nutrition ESPEN, Science Direct, 2022; pp 212–217. 10.1016/j.clnesp.2022.05.015 35871926

[12] Vasquez-Rojas, W. V. ; Martin, D. ; Miralles, B. Composition of Brazil Nut (Bertolletia Excels HBK), its Beverage and By-Products: A Healthy Food and Potential Source of Ingredients; Institute Food Science Research, 2021. 10.3390/foods10123007 PMC8700994 34945560

[13] Fernández-Ríos A., Battle-Bayer L., Azarkamand S. (2024). Development and application
of a nutritional quality model for life cycle assessment of protein-rich
foods. Sustainable Prod. Consump..

[14] Guo D., Liu C., Zhu H. (2024). Large edible mushrooms
and mycelial proteins:
A sustainable, nutritious protein source with health benefits and
processing innovations. Inovate Food Sci. Emerging
Technol..

[15] Santos A. M. P., Lima J. S., Santos I. F. (2019). Mineral and centesimal
composition evaluation of conventional and organic cultivars sweet
potato (Ipoema batatas L.) using chemometric tools. Food Chem..

[16] Official Methods of Analysis of the AOAC; AOAC International Publication: WA, 1984.

[17] Official Methods of Analysis of the AOAC; AOAC International Publications: WA, 1995.

[18] Barison A., Silva C. W. P., Campos F. R. (2010). A simple methodology
for the determination of fatty acid composition in edible oils through
H’NMR spectroscopy. Magn. Reson. Chem..

[19] Jham G. N., Teles F. F. F., Campos L. G. (1982). Use of aqueous HCl/MeOH as Esterification
Reagent for Analysis of Fatty Acids Derived from soybean lipids. J. Am. Oil Chem. Soc..

[20] da
Cruz Ferreira R., de Souza Dias F., de Aragão Tannus C. (2021). Essential and Potentially Toxic Elements from Brazilian Geopropolis
Produced by the Stingless Bee Melipona quadrifasciata anthidioides
Using ICP OES. Biol. Trace Elem Res..

[21] de
Aragão Tannus C., de Souza Dias F., Santana F. B. (2021). Multielement
Determination in Medicinal Plants and Herbal Medicines Containing
Cynara scolymus L., Harpagophytum procumbens D.C., and Maytenus ilifolia
(Mart.) ex Reiss from Brazil Using ICP OES. Biol. Trace Element Res..

[22] Gouveia S. T., Silva F. V., Costa L. M., Nogueira A. R. A., Nóbrega JÁ. (2001). Determination
of residual carbon by inductively-coupled plasma optical emission
spectrometry with axial and radial view configurations. Anal. Chim. Acta.

[23] White J. A., Hart R. J., Fry J. C. (1986). Na evaluation of
the Waters Pico-Tag
system for de amino-acid analysis of food materials. J. Automatic Chem..

[24] Kim D.-O., Chun O. K., Kim Y. J., Moon H. Y., Lee C. Y. (2003). Quantification
of polyphenolics and their antioxidant capacity in fresh plums. J. Agric. Food Chem..

[25] Sambrook, J. ; Fritsch, E. F. ; Maniatis, T. Molecular Cloning: A Laboratory Manual; Cold Spring Harbor Laboratory Press: New York, 1989.

[26] Yaping D., Xuli L., Ruyi L. (2024). Effects of ultrasound
treatment on the structure, function properties and in vitro digestion
of Sipunculus nudus protein. Int. J. Biol. Macromol..

[27] Isola, M. ; Colucci, G. ; Diana, A. Thermal Properties and decomposition products of modified cotton fibers by TGA, DSC, and Py-GC/MS. In Polym. Degrad. Stab.; Science Direct, 2024; p 228.

[28] Singh, A. ; Dhami, H. S. ; Sinha, M. K. Evaluation and comparison of mineralogical, micromeritics and rheological Properties of waste machining chips, coal fly ash particulates with metal and ceramic powders. In Powder Technol.; Science Direct, 2022; p 408.

[29] Wen M., Gao X., Liu Y. (2024). Prepatin of surface morphology of electrospun
nanofibers for biomedical applications. Acta
Mater. Compos. Sin..

[30] Grundl, G. Salting-out and salting-in effects of organic compounds and applications of the salting-out effect of Pentasodium phytate in different extraction processes. In J. Mol. Liq.; Science Direct, 2017; pp 368–375.

[31] Remígio M. S. N., Greco T., Júnior J. O. C.
S. (2024). Spray-drying
microencapsulation og Bauhinia ungulata L. var. obtusifolia Aqueous
Extract Containing Phenolic Compounds: A Comparative Study Using Different
Wall Materials. Pharmaceutics.

[32] Shao F., Zhang Y., Wan X. (2024). Regulation in protein
hydrophobicity via whey protein-zein self-assembly for improving the
techno-functional properties of protein. Food
Chem..

[33] Avila C. R., Hascoet A. S., Suárez J. V. M. (2019). Evaluation of the microbiological
contamination of food processing environments through implementing
surface sensors in na iberian pork processing plant> Na approach
towards
the control of Listeria monocytogenes. Food
Control..

[34] Yang J. (2009). Brazil nuts
associated health benefits: A review. Food Sci.
Technol..

[35] Balbi, M. E. ; Penteado, P. T. P. S. ; Cardoso, G. , Brazil nut (Bertholletia excelsa BONPL.) Composição Química e sua importância para saúde. 2014.

[36] Hatamnia A. A., Abbaspour N., Darvishzadeh R. (2014). Antioxidant activity
and phenolic profile of different parts of Bene (Pistacia atlantica
subsp. kurdica) fruits. Food Chem..

[37] Nasir, Y. ; Rahimi, M. H. Effect of omega-3 fatty acids supplementation on inflammatory markers following exercise-induced muscle damage: Systematic review and meta analysis of randomized controlled trials. In Nutrition Clinic et Metabolisme; Science Direct, 2024.

[38] Mohammadi T. (2024). Ameliorative
effects of omega-3 and omega-6 on spermatogenesis, testicular antioxidant
status and in vivo fertility index in heat-stressed rats. J. Thermal Biol..

[39] Laboratório Mont’Serrat (accessed: 20/09/2025).

[40] Alcântara D. B., Dionísio A. P., Artur A. G. (2022). Selenium
in Brazil nuts:
An overview of agronomical aspects, recent trends in analytical chemistry,
and health outcomes. Food Chem..

[41] National Academies of Sciences (NAS) . Dietary Reference Intakes for Vitamin C, Vitamin E, Selenium, and Carotenoids National Academy Press: Washington, DC, 2000. 25077263

[42] Luz G. M., Orlando E. A., Rebellato A. P. (2024). Essential minerals and
anti-nutritional compounds in plant-based burgers using the infogest
in vitro digestion protocol. J. Food Compos.
Anal..

[43] Tsunoda, M. ; Tsuda, T. Quantification of amino acids in small volumes of palm sweat samples. In Heliyon; Science Direct, 2024. 10.1016/j.heliyon.2024.e36286 PMC11387235 39263123

[44] Teng P. O.
Y., Choi J., Yadav S. (2021). Effects of low-crude
protein diets supplemented with arginine, glutamine, threonine, and
methionine on regulating nutrient absorption, intestinal health, and
growth performance of Eimeria-infected chickens. Poultry Sci..

[45] Barboza, L. P. ; Pereira, M. T. ; Fuzinatto, M. M. Caracterização Físico-química, antioxidante e de aminoácidos da castanha do baru, castanha de caju e castanha-do-brasil Qualidade de Produtos de Origem Animal 2019.

[46] Beutheu S., Ghouzali I., Galas L. (2013). Glutamine and arginine
improve permeability and tight junction protein expression in methotrexate-treated
Caco-2 cells. Clin Nutr..

[47] Cardoso, M. A. ; Scagliusi, F. B. Nutrição e Dietética; 2^a^ Edição, Guanabara Koogan, 2019.

[48] Cao T.-q., An H. X., Ma R. J. (2023). Structural characteristics
of a low molecular weigth velvet antler protein and the anti-tumor
activity on S180 tumor-bearing mice. Bioorg.
Chem..

[49] Sathe S. K., Venkatachalam M., Sharma G. M. (2009). 2024.
Solubilization
and Electroproretic Characterization of Select Edible Nut Seed Proteins. J. Agric. Food Chem..

[50] Xu T., Na J., Liu Q. (2024). The function of albumin and its application
in tumor therapy. Mater. Today Commun..

[51] Ruíz-Henestrosa V. P., Sánchez C. C., Escobar M. M. Y. (2007). Interfacial and foaming
characteristics of soy globulins as a function of pH and ionic strength. Sci. Direct.

[52] Liu H., Bai L., Jiang X. (2024). Recent progress in total synthesis of cyclic
peptides. Tetrahedron Lett..

[53] Koidl L., Gentile S. A., Untersmayr E. (2023). Allergen Stability
in Food Allergy:
A Clinician’s Perspective. Curr. Allergy
Asthma Rep..

[54] Chen Y., Li T., Jiang L. (2024). The composition, extraction, functional property,
quality, and health benefits of coconut protein: A review. Int. J. Biol. Macromol..

[55] Shao F., Zhang Y., Wan X. (2025). Regulation in protein
hydrophobicity via whey protein-zein self-assembly for improving the
techno-functional Properties of protein. Food
Chem..

[56] Mota A. M., Carvalho M. da S., das
Virgens C. F. (2022). Enriquecimento de Pectina através
da técnica squeeze-flow empregando a concha da Lucina Pectinata
como fonte de carbonato de cálcio. J.
Eng. Exact Sci..

[57] Camelo E. R., Castro J. D. S., das Virgens C. F. (2023). Thermokinetic evaluation of zircon
oxide green synthesis mediated by plant extract of Abelmoschus esculentus
L. Moench. J. Therm. Anal. Calorim..

[58] das
Virgens C. F., Castro J. D. S. (2021). Screening of slow pyrolysis routes
for maximum biofuel production from Syzygium malaccense biomass by
TGA-FSD and chemometric tools. J. Therm. Anal.
Calorim..

[59] Li K., Woo M. W., Patel H. (2018). Improvement of rheological
and functional Properties of milk protein concentrate by hydrodynamic
cavitation. J. Food Eng..

[60] Tran D. T., Majerová D., Vesely M. (2019). On the mechanism of
colloidal sílica action to improve flow properties of pharmaceutical
excipients. Int. J. Pharm..

[61] Dias, I. L. T. ; Zanotti, A. C. ; Crevelin, C. A. Desenvolvimento tecnológico de cápsulas contendo paracetamol granulado. In Rev. Eletrôn. Farmácia.; Universidade Federal de Goiás, 2012; pp 1–19.

[62] Moreno A. d. H., Abreu M. C. (2019). Estudo comparativo de quatro métodos farmacotécnicos
para preenchimento de cápsulas gelatinosas rígidas. Rev. Bras. Multidiscip..

[63] Cury, B. S. F. ; Silva Junior, N. P. ; Castro, A. D. Influência das propriedades de granulados de celulose nas características físicas dos comprimidos Rev. Ciências Farmacêuticas Básica Aplicada. 2008.

[64] Meram A., Ismail T. (2024). Physical, chemical, and technological properties of
protein isolate obtained from hazelnut press cake as affected by drying
technique. Food Biosci..

[65] Gul O., Gul L., Baskinci T. (2023). Influence of pH and
ionic strength on
the bulk and interfacial rheology and technofunctional properties
of hazelnut meal protein isolate. Food Res.
Int..

[66] Ogunwolo S. O., Henshaw F. O., Mock H. P. (2009). Functional
properties of protein
isolate concentrates and isolates produced from cashew (Anacardium
occidentale L.) nut. Food Chem..

[67] Deng L. (2021). Current Progress
in the Utilization of Soy-Based Emulsifiers in Food Applications—A
Review. Foods.

[68] Grisham, D. R. ; Nanda, V. Zeta Potential Prediction from Protein Structure in General Aqueous Eletrolyte solutions American Chemical Society, 2020; pp 13799–13803. 10.1021/acs.langmuir.0c02031 33186035

[69] Renkema J. M. S., Gruppen H., Vliet T. V. (2002). Influence of pH
and Ionic Strength
on Heat-Induced Formation and Rheological Properties of Soy Protein
Gels in Relation to Denaturation and Their Protein Compositions. J. Agric. Food Chem..

