# Supplementary material for: Production and Characterization of a Brazil Nut Protein Isolate with Functional Properties for Food Applications
Source: ACS Omega. 2026 Sep 1;11(36):54041–52. doi: 10.1021/acsomega.6c04239 (PMC13584518; doi:10.1021/acsomega.6c04239)
Supplement: Supplementary file 1 [file ao6c04239_si_002.pdf]

Production and Characterization of a Brazil Nut Protein Isolate with Functional Properties for Food Applications

João Lucas da Silva Rufino[1], Pâmela Vital da Silva de Assunção[1], Laura Beatriz Souza e Souza[2], João Pedro Cezário Brandão[2], Betina Raquel de Oliveira Araújo[3], Keyla Emanuelle Ramos de Holanda[1], Aníbal de Freitas Santos Junior[2], Edmar Vaz de Andrade[3], Emersom Silva Lima[1*]

[1]Faculdade de Ciências Farmacêuticas (FCF), Universidade Federal do Amazonas (UFAM) Av. Gen. Rodrigo Otavio, nº 6200, Coroado, CEP 69077-000. Manaus-AM, Brazil.

[2]Departamento de Ciências da Vida (DCV), Universidade Estadual da Bahia (UNEB), Rua Silveira Martins, nº 2555, Cabula, Salvador - BA, CEP: 41.150-000

[3]Instituto de Ciências Biológicas (ICB), Universidade Federal do Amazonas (UFAM). Av. Gen. Rodrigo Otavio, nº 6200, Coroado, CEP 69077-000. Manaus-AM, Brazil.

Corresponding Author

Emersom Silva Lima – Faculty of Pharmaceutical Sciences; Federal University of Amazonas – UFAM. Manaus/AM 69080-900, Brazil; https://orcid.org/0000-0002-9367-2812 Email: eslima@ufam.edu.br

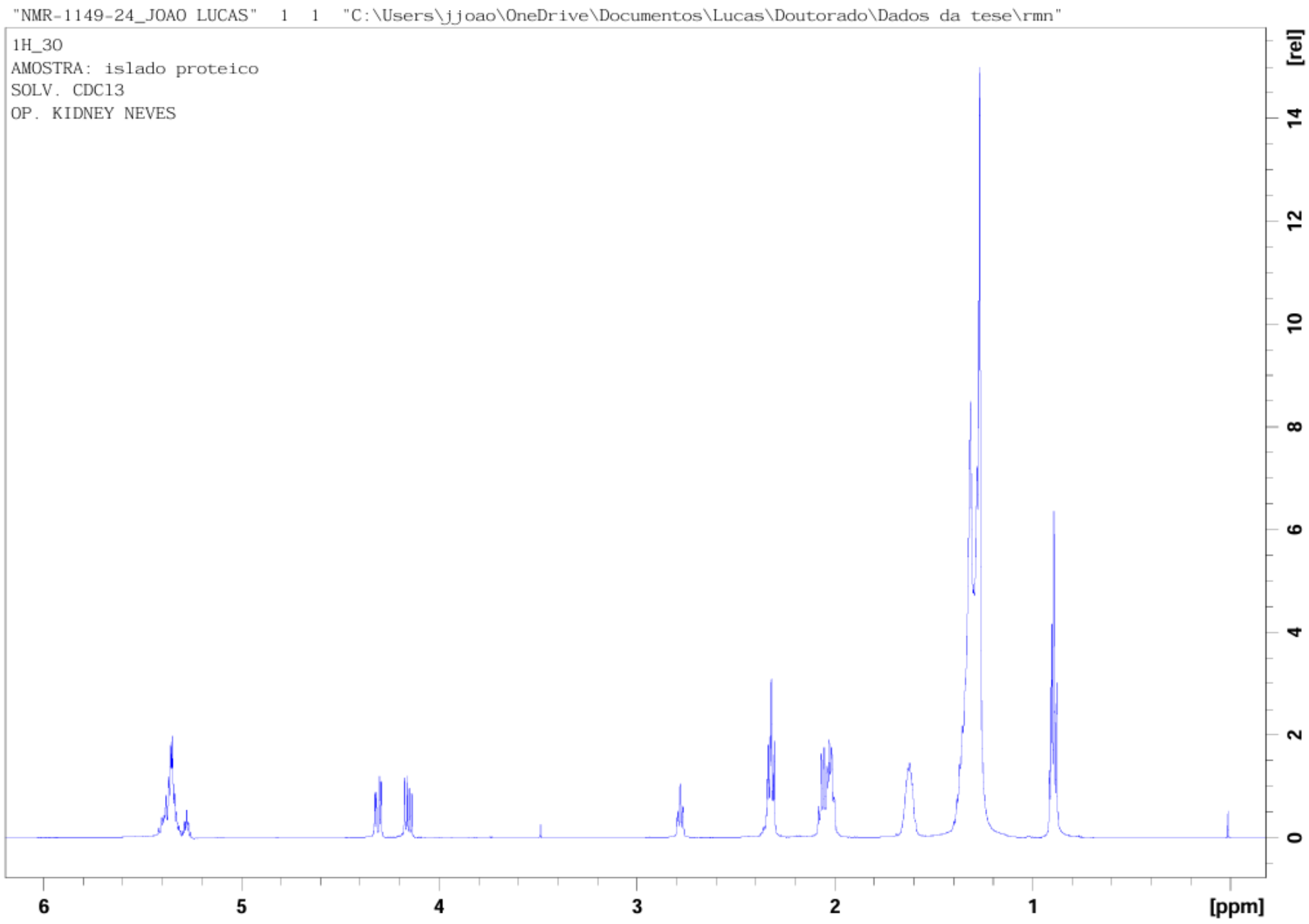
"NMR-1149-24_JOAO LUCAS" 1 1 "C:\Users\jjoao\OneDrive\Documentos\Lucas\Doutorado\Dados da tese\rmn"
1H_30
AMOSTRA: islado proteico
SOLV. CDC13
OP. KIDNEY NEVES
[rel]
14
12
10
8
6
4
2
0
6
5
4
3
2
1
[ppm]

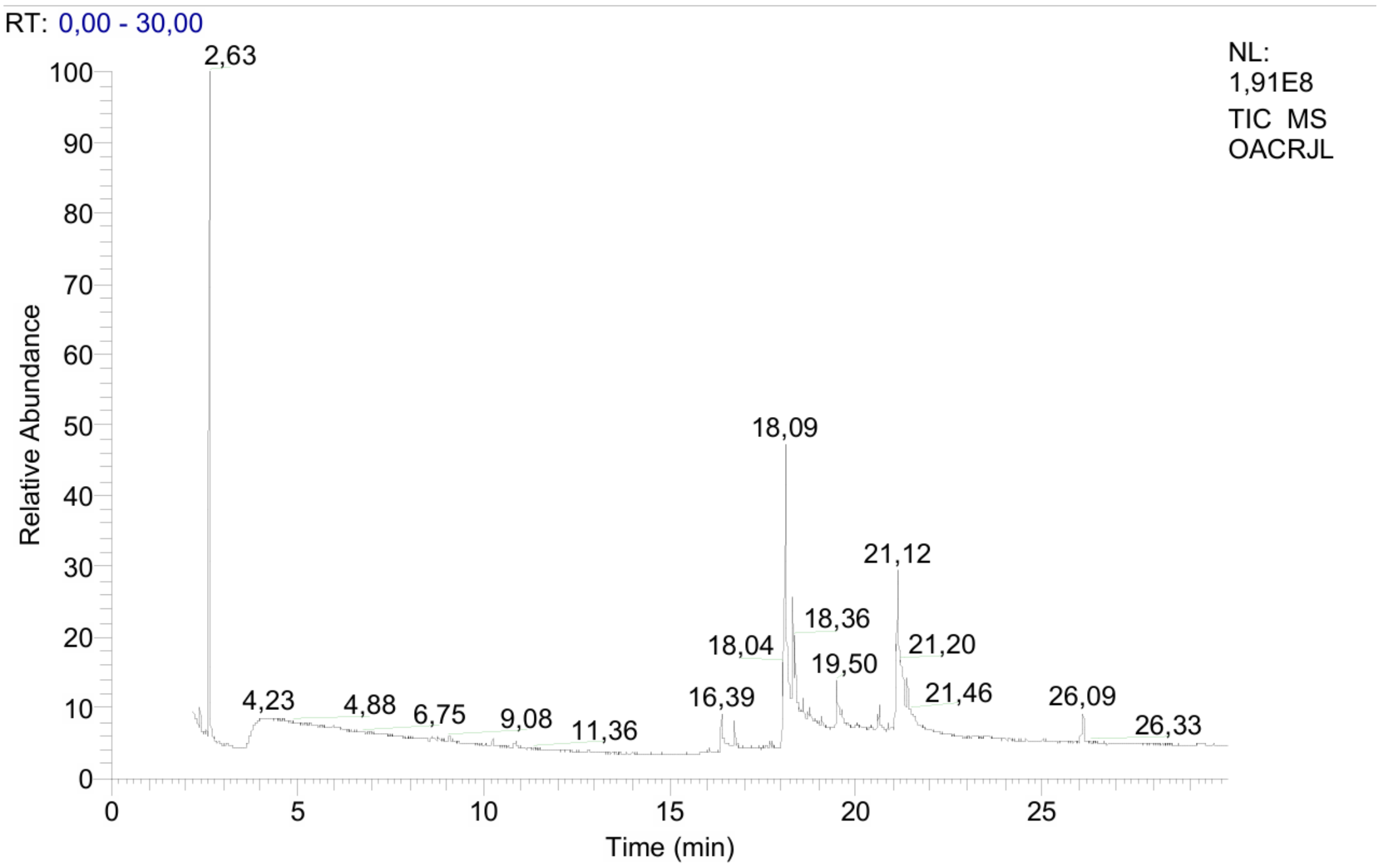

RT: 0,00 - 30,00
NL:
1,91E8
TIC MS
OACRJL
Relative Abundance
Time (min)
2,63
4,23
4,88
6,75
9,08
11,36
16,39
18,04
18,09
18,36
19,50
21,12
21,20
21,46
26,09
26,33
